# Clinical Considerations of Splenic Dose Constraints to Mitigate Radiation‐Induced Lymphopenia

**DOI:** 10.1002/cam4.71553

**Published:** 2026-01-22

**Authors:** Yifu Ma, Shuying Zhang, Jiayan Ma, An Gao, Jiale Liu, He Ma, Qiyi Zhou, Jianjun Qian, Liyuan Zhang

**Affiliations:** ^1^ PRaG Therapy Center, Center for Cancer Diagnosis and Treatment The Second Affiliated Hospital of Soochow University Suzhou China; ^2^ Department of Radiotherapy and Oncology The Second Affiliated Hospital of Soochow University Suzhou China; ^3^ Institute of Radiotherapy and Oncology Soochow University Suzhou China; ^4^ Department of Ultrasound The Second Affiliated Hospital of Soochow University Suzhou China; ^5^ Department of Experimental Center The Second Affiliated Hospital of Soochow University Suzhou China; ^6^ State Key Laboratory of Radiation Medicine and Protection Soochow University Suzhou China

**Keywords:** lymphocyte recovery index, lymphopenia, radiotherapy, spleen

## Abstract

**Background:**

The spleen dose‐volume threshold for lymphopenia in abdominal radiotherapy has not yet reached a consensus. Our previous research indicated a correlation between these factors, but the threshold has not been determined. Therefore, we investigated the dynamic changes in lymphocytes during radiotherapy (RT), identified the spleen dose threshold, and examined how these factors affect patient prognosis.

**Methods:**

The absolute lymphocyte counts (ALC) of gastric cancer patients were collected before, during, and after RT. Lymphocyte recovery status was assessed using the lymphocyte recovery index (LRI). LRI cut off was considered as insufficient recovery. Splenic dosimetric parameters were collected, and their impact on predicting grade 4 (G4) lymphopenia was evaluated using logistic regression analysis. Cox regression analysis was used to evaluate the relationship between lymphocyte depletion and recovery status and prognosis.

**Results:**

159 patients were enrolled. The median ALC dropped by 85.71% after RT. The occurrence of G4 and G1‐3 lymphopenia was observed in 30.2% and 69.8% of cases, respectively. There were 12.6% of patients whose ALC had recovered at 120 days after RT, while the remaining 87.4% were still accompanied by lymphopenia. Cox multivariable analysis showed that pTNM stage and LRI were independent prognostic factors affecting overall survival, and the independent prognostic factors for disease‐free survival were pTNM stage and change in ALC. Splenic D_mean_ and V_5_ were related to G4 lymphopenia and eventually V_5_ affected prognosis. Constraining the spleen V_5_ to < 180.6 cm^3^ and < 272.2 cm^3^ may reduce the incidence of G4 lymphopenia and further decrease the risk of death by 60.9%.

**Conclusions:**

Patients with severe lymphocyte decline during RT and insufficient lymphocyte recovery afterward have a worse prognosis. It is important not only to prevent severe lymphopenia during RT but also to focus on improving lymphocyte recovery after RT. Constraining the spleen V_5_ is a key approach.

**Abbreviations**
ALC: Absolute lymphocyte count
ANC: Absolute neutrophil count
AUC: Area under the curve
CAPOX: Capecitabine and oxaliplatin
CI: Confidence interval
CRT: Chemoradiotherapy
CTCAE: Common terminology criteria for adverse events
DFS: Disease‐free survival
EGJ: Esophagogastric junction
EOF: Epirubicin, oxaliplatin, and fluorouracil
FOLFOX: Folinic acid, fluorouracil, and oxaliplatin
GC: Gastric cancer
HR: Hazard ratio
LNs: Lymph nodes
LRI: Lymphocyte recovery index
MVA: Multivariable analyses
NSCLC: Non‐small cell lung cancer
OAR: Organ at risk
OR: Odds ratio
OS: Overall survival
PET: Positron emission tomography
RIL: Radiation‐induced lymphopenia
ROC: Receiver operating characteristic
SOX: S‐1 and oxaliplatin
UVA: Univariable analyses
WBC: White Blood cell count

## 1 Introduction

Radiotherapy (RT) is one of the main treatments for cancer, and it acts as a double‐edged sword. While it helps to eliminate tumors, it also leads to the loss of lymphocytes. Lymphocytes are highly sensitive to radiation, and radiation‐induced lymphopenia (RIL) is considered a common complication after RT, with an overall incidence ranging from 40% to 70% [1, 2]. Several studies on solid tumors have shown that the occurrence of RIL is associated with poor prognosis, and insufficient recovery of lymphocytes also indicates a worse outcome [3, 4]. Lymphocytes are crucial for immune therapy, and with the increasing use of combination therapies involving RT and immunotherapy in clinical practice, the depletion and recovery of lymphocytes during RT have gained more attention.

The spleen, bone marrow, and thymus are rich in lymphoid and hematopoietic tissues. Among them, the spleen is the largest secondary lymphoid organ, containing approximately 15% of the body's lymphocytes. Over 90% of the blood in the body circulates through the spleen, while the number of lymphocytes in circulating blood is only about one‐seventh of that in the spleen [5]. Additionally, the spleen serves as the site for B lymphocyte responses and T cell activation, playing a crucial role in both innate and adaptive immunity [6].

Irradiation of the spleen is a significant cause of lymphopenia, and the spleen is rarely intentionally irradiated and has rarely been considered an organ at risk (OAR), although it is often exposed during radiotherapy for thoracoabdominal tumors. Therefore, investigating the relationship between RIL and spleen irradiation is crucial, and some studies have explored this relationship in recent years [7, 8, 9]. Our previous study in April 2024 described that spleen V_5_ was an independent predictor of minimum absolute lymphocyte count (ALC) and that lymphocyte decline is associated with a worse prognosis [10], but there was a lack of dynamic changes in ALC during and after radiotherapy and quantitative evidence of spleen dose constraints, while the impact of recovery from lymphopenia on prognosis was controversial in previous studies [11, 12, 13].

Therefore, based on previous studies, this research expanded the patient sample size and further analyzed the recovery status of lymphocytes. The aim is to investigate the relationship between lymphocyte reduction and recovery during chemoradiotherapy (CRT) and patient prognosis, as well as to further quantify the spleen dose constraints that impact lymphopenia.

## 2 Methods

### 2.1 Patient Selection

Due to the heterogeneity of abdominal malignant tumors and the fact that the radiotherapy target area for gastric cancer (GC) typically includes the spleen, we conducted a retrospective analysis of GC patients who underwent postoperative CRT at our hospital between January 2010 and January 2020. Patients who had received preoperative treatment, had distant metastasis before surgery, or did not receive standard radical surgery were excluded. Patients who did not undergo standard radical surgery were defined as those without an R0 gastrectomy and D1+ lymphadenectomy or higher. These patients were excluded because insufficient lymph node dissection may affect treatment planning and prognosis. Since most gastric cancer patients at our center received R0/D1+ radical resection as the standard procedure, the number of excluded cases was very limited, and the potential selection bias is considered minimal. The Karnofsky Performance Status (KPS) score was used to evaluate patients' functional status, while serum albumin levels were measured to evaluate their nutritional status.

### 2.2 Treatment Planning

All patients were scheduled to receive intensity‐modulated radiotherapy with a total dose of 45.0–50.4 Gy, delivered in 25–28 fractions (5 days/week, 1.8–2.0 Gy/day). The selection of regional lymph nodes was based on the tumor location (Table S1). Dose constraints included a maximum spinal cord dose ≤ 45 Gy, kidney V_20_ < 25%, liver V_30_ < 30%, kidney V_15_ < 60%, and heart V_30_ < 30%. The spleen was not subject to any dose constraints. The contouring of the spleen was performed by radiation oncologists for each patient, and dose calculations and approvals were conducted by medical physicists using the Pinnacle 9.0 treatment planning system (Philips Radiation Oncology Systems, Fitchburg, WI, USA). Dose‐volume histogram analysis was used to determine the mean dose (D_mean_), maximum dose (D_max_), and absolute volume of the spleen receiving 5, 10, 15, 20, 25, 30, 35, 40, and 45 Gy (V_5_, V_10_, V_15_, V_20_, V_25_, V_30_, V_35_, V_40_, and V_45_), respectively.

Concurrent chemotherapy regimens included capecitabine, S‐1 (Tegafur/Gimeracil/Oteracil), and other agents. All enrolled patients received adjuvant chemotherapy either before or after radiotherapy. The chemotherapy regimens included CAPOX (capecitabine and oxaliplatin), SOX (S‐1 and oxaliplatin), FOLFOX (leucovorin, fluorouracil, and oxaliplatin), EOF (epirubicin, oxaliplatin, and fluorouracil), and other agents.

### 2.3 Assessment of Absolute Lymphocyte Count

The most recent ALC before CRT was recorded as a baseline, and ALC were collected weekly during CRT and at 60, 90, and 120 (± 10) days post‐CRT. According to Common Terminology Criteria for Adverse Events 5.0 (Grade 1: ALC < 0.8 × 10^9^/L; Grade 2: 0.8–0.5 × 10^9^/L; Grade 3: 0.5–0.2 × 10^9^/L; Grade 4: < 0.2 × 10^9^/L), grade 4 (G4) lymphopenia is defined as ALC nadir < 0.2 × 10^9^/L during CRT. Lymphopenia is defined as ALC < 1.1 × 10^9^/L based on the lower normal limit of ALC in our institution. Lymphocyte recovery capacity was estimated using the lymphocyte recovery index (LRI, Equation 1), which was defined as the ratio of posttreatment to baseline lymphocyte counts. The ability to resist lymphocyte depletion during CRT was assessed based on the change in ALC (ΔALC, Equation 2).

(1) $$\mathrm{LRI}=\frac{\mathrm{ALC}\;\mathrm{at}\;120\;\text{days after}\;\mathrm{CRT}}{\mathrm{ALC}\;\mathrm{at}\;\text{baseline}}$$

(2) ΔALC=ALCatbaseline−ALCnadir duringCRT

### 2.4 Patient Grouping

The receiver operating characteristic (ROC) curves based on overall survival (OS) were utilized to determine the optimal cutoff values for LRI and ΔALC. Patients with LRI < the cutoff value were classified as having insufficient lymphocyte recovery (LRI^lo^), while those with LRI ≥ the cutoff value were classified as having adequate lymphocyte recovery (LRI^hi^). The population was then divided into four groups based on whether ΔALC was below the cutoff value: (1) Group A (ΔALC^hi^ LRI^lo^); (2) Group B (ΔALC^hi^ LRI^hi^); (3) Group C (ΔALC^lo^ LRI^lo^); and (4) Group D (ΔALC^lo^ LRI^hi^).

### 2.5 Follow‐Up

Routine follow‐up after adjuvant CRT treatment includes a review of medical history, physical examination, blood tests (serum biochemical markers and tumor markers), and CT scans (with positron emission tomography‐CT if necessary). For the first 2 years, patients are followed up every 3 months, then every 6 months until the fifth year, and annually thereafter.

### 2.6 Statistical Analysis

Descriptive statistics were used to present clinical variables and dosimetric parameters, with group differences assessed using the Mann–Whitney *U* test or Chi‐squared tests. Univariable (UVA) and multivariable (MVA) logistic regression analyses evaluated the impact of variables on predicting G4 lymphopenia, with cross‐correlation among dosimetric parameters tested prior to logistic regression. The correlation between variables and survival outcomes was analyzed using Cox proportional hazards modeling and the Kaplan–Meier method with log‐rank tests. Backward elimination was applied to derive the final model in multivariable analysis. ROC curves determined the ideal thresholds for splenic V_5_, LRI, and ΔALC, with the area under the curve (AUC) used to assess predictive performance. The optimal cutoff for V_5_ was identified using the maximally selected log‐rank statistics method based on OS. Statistical analyses were conducted using SPSS Statistics 25.0 (IBM Corp., Armonk, NY, USA) and GraphPad Prism 8.0 (GraphPad Software, San Diego, CA, USA), with statistical significance set at *p* < 0.05 (two‐sided tests).

## 3 Results

### 3.1 Patient Characteristics and ALC Data

This study retrospectively analyzed 159 eligible patients (Figure S1), including 120 (75.5%) males and 39 (24.5%) females. The median age of the enrolled patients was 62 years. In terms of cancer staging, 48 patients (30.2%) were at stage II, while 109 patients (68.5%) had tumors at stage III. A total of 95 patients (59.7%) received capecitabine, 24 patients (15.1%) were treated with S‐1, and 40 patients (25.2%) received other agents during RT. The adjuvant chemotherapy regimens included CAPOX (89, 56.0%), SOX (19, 11.9%), EOF (7, 4.4%), FOLFOX (13, 8.2%), and other medications (31, 19.5%). All patients underwent adjuvant chemotherapy before or after RT. Of the 159 patients, 6 did not complete the radiation therapy regimen. The clinical characteristics of the enrolled patients are detailed in Table 1.

**TABLE 1:** The clinical and dosimetric data of 159 gastric cancer patients.

Characteristics	All patients (*n* = 159)	Patients categorized by ALC during CRT		
		ALC ≥ 0.2 × 10^9^/L (*n* = 111)	ALC < 0.2 × 10^9^/L (*n* = 48)	*p*
Age (year), median (range)	62 (27–87)	62 (27–87)	62 (33–77)	0.990
Sex, *n* (%)				0.756
Male	120 (75.5)	83 (74.8)	37 (77.1)	
Female	39 (24.5)	28 (25.2)	11 (22.9)	
Stage^a^, *n* (%)				0.641
I	2 (1.3)	2 (1.8)	0 (0.0)	
II	48 (30.2)	33 (29.7)	15 (31.3)	
III	109 (68.5)	76 (68.5)	33 (68.7)	
Concurrent chemotherapy regimen, *n* (%)				0.431
Capecitabine	95 (59.7)	63 (56.8)	32 (66.7)	
S‐1	24 (15.1)	19 (17.1)	5 (10.4)	
Others	40 (25.2)	29 (26.1)	11 (22.9)	
Adjuvant chemotherapy regimen, *n* (%)				0.886
CAPOX	89 (56.0)	63 (56.8)	26 (54.2)	
SOX	19 (11.9)	14 (12.6)	5 (10.4)	
FOLFOX	7 (4.4)	4 (3.6)	3 (6.3)	
EOF	13 (8.2)	8 (7.2)	5 (10.4)	
Others	31 (19.5)	22 (19.8)	9 (18.7)	
WBC, median (range) (10^9^/L)				
Before chemoradiation	5.0 (2.0–22.4)	5.0 (2.0–22.4)	5.1 (2.4–17.8)	0.223
After chemoradiation	3.5 (0.8–29.0)	3.6 (0.8–29.0)	3.2 (1.2–12.8)	0.449
ALC, median (range) (10^9^/L)				
Before chemoradiation	1.4 (0.2–3.7)	1.4 (0.2–3.7)	1.45 (0.3–2.7)	0.907
After chemoradiation	0.5 (0–2.0)	0.5 (0–1.9)	0.2 (0–2.0)	**0.001**
ANC, median (range) (10^9^/L)				
Before chemoradiation	3.0 (1.0–19.8)	2.8 (1.0–19.8)	3.0 (1.1–15.2)	0.167
After chemoradiation	2.15 (0.5–27.6)	2.2 (0.5–27.6)	2.1 (0.8–12.3)	0.816
Maximum spleen dose (Gy), median (range)	4879.0 (232.0–6040.0)	4860.0 (232.0–6040.0)	4913.5 (3117.0–5696.0)	**0.037**
Mean spleen dose (Gy), median (range)	2594.0 (15.6–4139.0)	2478.0 (15.6–3962.0)	2745.0 (1177.0–4139.0)	**0.049**
Spleen V5 (cm^3^), median (range)	173.31 (0.00–573.70)	159.63 (0.00–506.45)	211.71 (61.50–573.70)	**0.000**
Spleen V10 (cm^3^), median (range)	153.68 (0.00–1418.68)	144.70 (0.00–1418.68)	182.23 (61.50–542.89)	**0.000**
Spleen V15 (cm^3^), median (range)	137.87 (0.00–463.64)	127.12 (0.00–421.38)	167.80 (59.31–463.64)	**0.000**
Spleen V20 (cm^3^), median (range)	118.90 (0.00–356.00)	106.04 (0.00–356.00)	152.69 (47.78–343.10)	**0.000**
Spleen V25 (cm^3^), median (range)	96.81 (0.00–275.95)	74.01 (0.00–275.95)	127.40 (6.25–268.75)	**0.000**
Spleen V30 (cm^3^), median (range)	69.76 (0.00–219.85)	56.31 (0.00–219.85)	95.58 (0.00–205.30)	**0.000**
Spleen V35 (cm^3^), median (range)	43.41 (0.00–153.70)	37.83 (0.00–144.48)	58.65 (0.00–153.70)	**0.001**
Spleen V40 (cm^3^), median (range)	28.32 (0.00–124.77)	25.05 (0.00–124.77)	36.31 (0.00–113.81)	**0.003**
Spleen V45 (cm^3^), median (range)	13.09 (0.00–104.56)	11.14 (0.00–104.56)	17.57 (0.00–58.76)	**0.004**

Abbreviations: ALC, absolute lymphocyte count; ANC, absolute neutrophil count; CAPOX, capecitabine and oxaliplatin; CRT, chemoradiotherapy; EOF, epirubicin, oxaliplatin, and fluorouracil; FOLFOX, folinic acid, fluorouracil, and oxaliplatin; SOX, S‐1 and oxaliplatin; Vx, volume of spleen receiving × Gy of radiation; WBC, white Blood cell count. Bold values indicate statistical significance (*p* < 0.05).

^a^ According to the 8th edition of the American Joint Committee on Cancer system.

**FIGURE 1:**
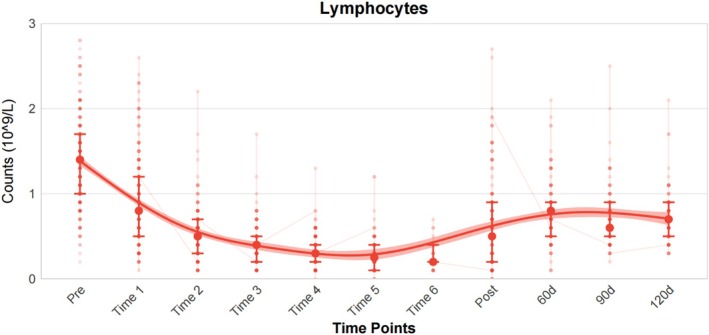
The dynamic changes of lymphocyte from baseline through week 1–6 and 120 days after the end of CRT, represented by median value with quartiles. *Pre* represented ALC at baseline. Time 1–6 represented ALC at week 1–6 during CRT. *Post* represented ALC at time just end of radiotherapy. 60 days, 90 days, and 120 days represented ALC at time 60 days, 90 days, and 120 days after CRT. ALC, absolute lymphocyte count; CRT, Chemoradiotherapy.

Visualization of the ALC for 159 patients revealed that during treatment, ALC decreased weekly, typically reaching its lowest point at week 6, and then gradually increased to near 1 × 10^9^/L levels (Figure 1). The median ALC values (×10^9^/L) at baseline and during weeks 1–6 of CRT were 1.4, 0.8, 0.5, 0.4, 0.3, 0.25, and 0.2, respectively. After completing CRT, the median ALC decreased by 85.71%. A total of 48 patients (30.2%) and 111 patients (69.8%) experienced G4 and G1‐3 lymphopenia, respectively. The dynamic changes of WBC and neutrophils can be found in Figure S2.

The recovery of ALC after RT is a lengthy and challenging process. A total of 20 patients (12.6%) had their ALC restored to normal levels (≥ 1.1 × 10^9^/L) 120 days after completing CRT, while the majority of patients (87.4%) still experienced varying degrees of lymphopenia. The number of patients with grade 1, 2 and 3 lymphopenia were 42 (26.4%), 69 (43.4%), and 28 (17.6%), respectively. All patients' median of ALC (× 10^9^/L) was 1.4 (range 0.3–2.1) at baseline, and 40 (25.2%) of them had G1‐3 lymphopenia. The median of ALC at 120 days after CRT was 0.70 × 10^9^/L (range 0.3–2.1).

### 3.2 Optimal Threshold of Splenic V_5_ for G4 Lymphopenia

**FIGURE 2:**
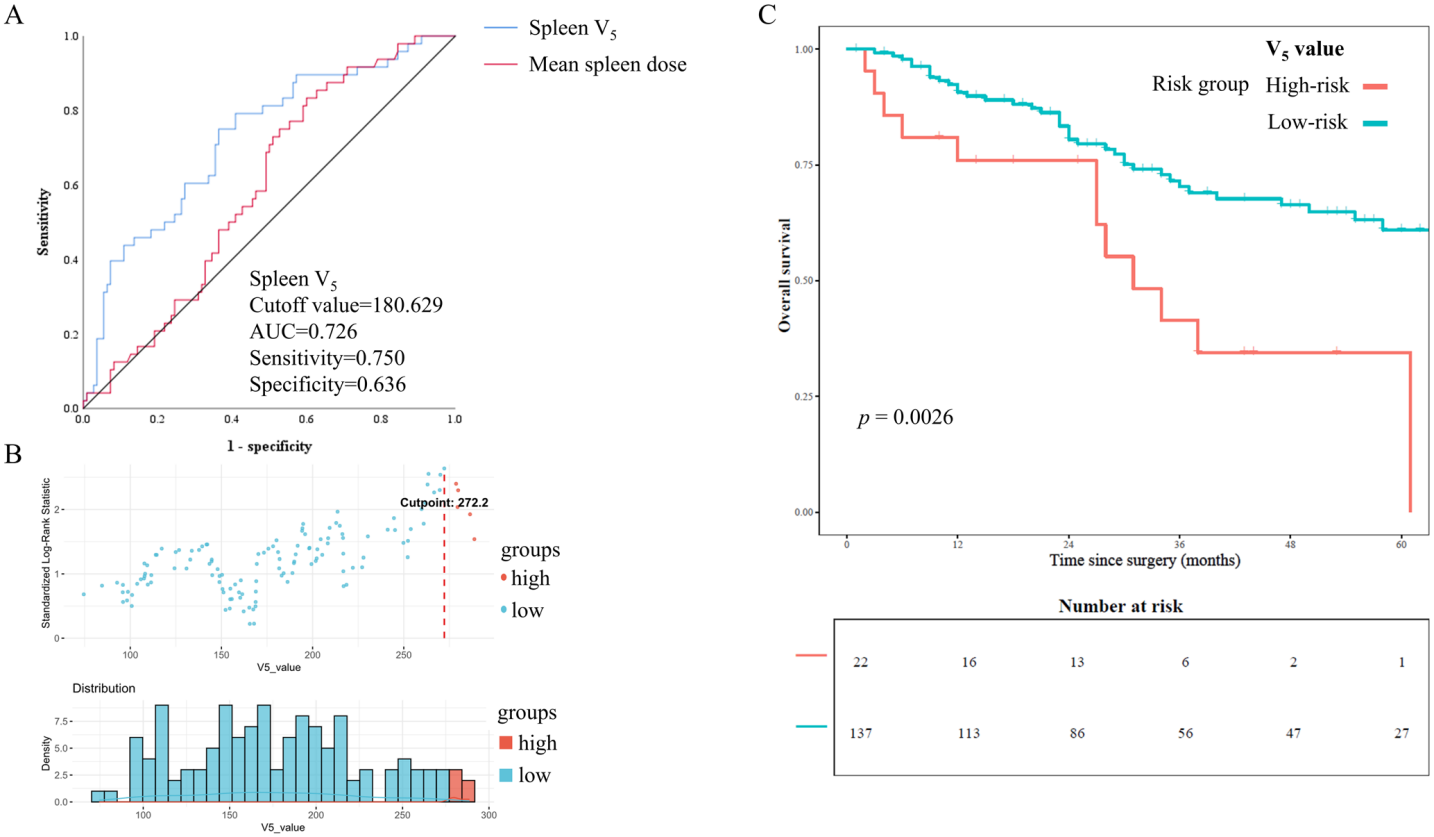
Optimal threshold of spleen V_5_ for (A) G4 lymphopenia, (B) overall survival. (C) Kaplan Meier curves of spleen V_5_ high‐risk group and low‐risk group (spleen V_5_ cutoff value = 272.2 cm^3^). *Time since surgery* refers to the time elapsed since R0/D1+ radical resection for gastric cancer in enrolled patients.

Univariate analysis results showed that spleen dosimetric parameters (except for D_max_ and V_10_) were associated with G4 lymphopenia. To eliminate the correlation between two adjacent dosimetric parameters (Figure S3, Table S2), we selected age, gender, pTNM stage, pre‐ALC, spleen D_mean_, and V_5_ in the multivariable analysis with backward elimination. Multivariate analysis showed that the D_mean_ and V_5_ were related to G4 lymphopenia (Table 2). To further evaluate whether the chemotherapy regimen modified the effect of splenic irradiation, we performed interaction analyses between splenic dose parameters and the chemotherapy regimen, both during and after CRT. None of the interaction terms reached statistical significance (all *p* > 0.05, Tables S3 and S4), indicating that the chemotherapy regimen did not significantly alter the association between splenic dose and G4 lymphopenia. As shown in Figure 2A, the optimal threshold for spleen V_5_ in predicting G4 lymphopenia was 180.6 cm^3^ (AUC = 0.726, sensitivity = 0.750, specificity = 0.636, *p* = 0.006). Further analysis revealed that limiting V_5_ to < 272.2 cm^3^ decreased the death risk by 60.9% (*p* = 0.0026) in Figure 2B,C.

**TABLE 2:** Univariable and multivariable analyses of grade 4 lymphopenia during CRT for the patients.

	UVA		MVA	
	OR (95% CI)	*p*	OR (95% CI)	*p*
Clinical characteristics				
Age (< 60/≥ 60, years)	1.198 (0.593–2419)	0.615		
Gender (female/male)	0.881 (0.397–1.957)	0.756		
pTNM stage (I/II/III)^a^	1.090 (0.546–2.173)	0.808		
Pre‐ALC (×10^9^/L)	0.890 (0.489–1.620)	0.702		
Dosimetric parameters				
Maximum spleen dose (Gy)	1.001 (1.000–1.002)	0.067		
Mean spleen dose (Gy)	1.001 (1.000–1.001)	**0.024**	1.001 (1.000–1.001)	**0.020**
Spleen V5 (cm^3^)	1.009 (1.005–1.013)	**0.000**	1.010 (1.005–1.014)	**0.000**
Spleen V10 (cm^3^)	1.004 (1.000–1.007)	0.053		
Spleen V15 (cm^3^)	1.012 (1.006–1.018)	**0.000**		
Spleen V20 (cm^3^)	1.014 (1.007–1.020)	**0.000**		
Spleen V25 (cm^3^)	1.014 (1.007–1.021)	**0.000**		
Spleen V30 (cm^3^)	1.014 (1.006–1.022)	**0.000**		
Spleen V35 (cm^3^)	1.015 (1.005–1.025)	**0.002**		
Spleen V40 (cm^3^)	1.018 (1.005–1.032)	**0.006**		
Spleen V45 (cm^3^)	1.022 (1.000–1.045)	**0.049**		

Abbreviations: CI, confidence interval; CRT, chemoradiotherapy; MVA, multivariable analyses; OR, odds ratio; UVA, univariable analyses; Vx, volume of spleen receiving × Gy of radiation. Bold values indicate statistical significance (*p* < 0.05).

^a^ According to the 8th edition of the American Joint Committee on Cancer system.

### 3.3 Survival Outcomes and Prognostic Factors

**FIGURE 3:**
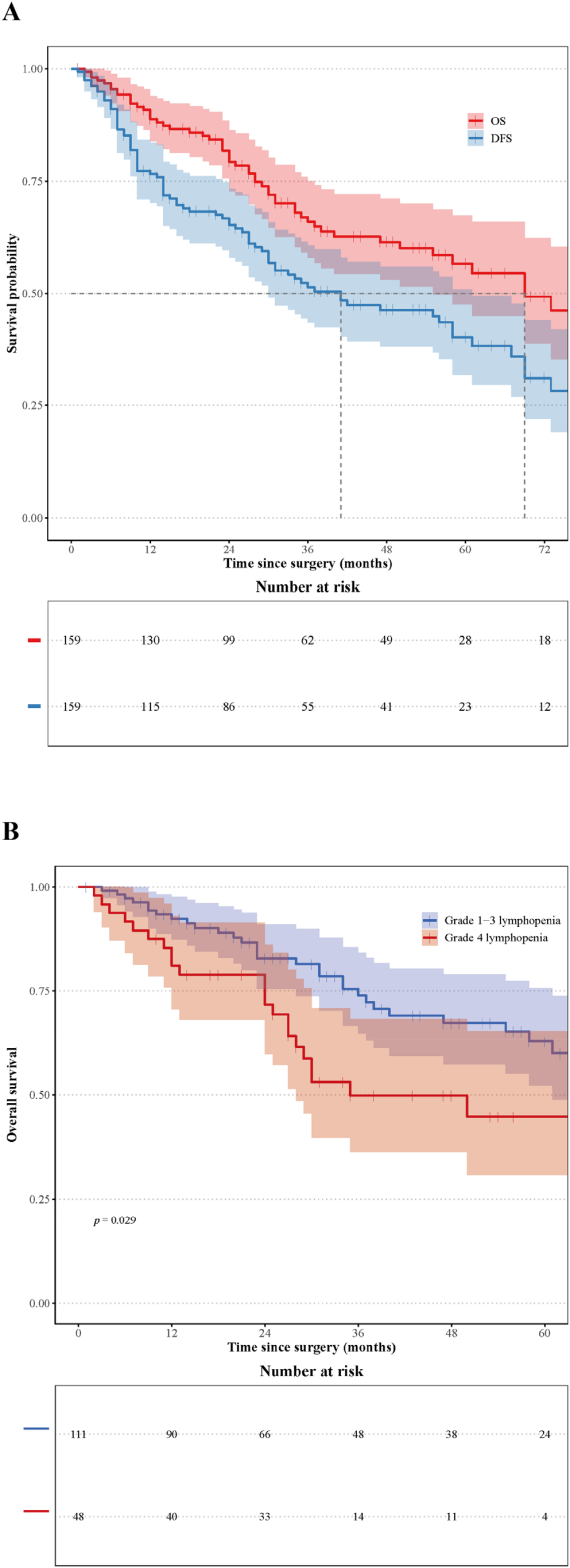
Kaplan Meier curves of (A) overall survival and disease‐free survival, (B) grade 1–3 lymphopenia and grade 4 lymphopenia patients' overall survival. Grade 4 lymphopenia is defined as absolute lymphocyte count < 0.2 × 10^9^/L during chemoradiotherapy. *Time since surgery* refers to the time elapsed since R0/D1+ radical resection for gastric cancer in enrolled patients. The shaded area represents the 95% confidence interval of the curve.

As of October 2024, the median follow‐up time for the enrolled patients was 48.0 months (range 1–130 months). A total of 56 patients (35.2%) have died, with a median OS time of 69.0 months. Forty‐nine patients (30.8%) experienced relapse at 56 sites (19 locoregional recurrence and 37 distant metastasis). The median disease‐free survival (DFS) time was 41.0 months. The 1‐, 3‐, and 5‐year OS were 88.80%, 65.93%, and 56.62%. The 1‐, 3‐, and 5‐year DFS were 76.62%, 51.36%, and 40.23%, respectively (Figure 3A). Univariable analysis indicated that pTNM stage, G4 lymphopenia, LRI, ΔALC, V_5_, V_15_, and V_20_ are factors influencing OS. Further multivariable analysis revealed that pTNM stage and LRI are independent prognostic factors (Table 3). Splenic V_5_‐V_45_ is always related to LRI (Table S5). Prognostic factors for DFS include pTNM stage, pre‐ALC, and ΔALC. Multivariable analysis showed that pTNM stage and ΔALC were independent prognostic factors related to DFS (Table 4).

**TABLE 3:** Univariable and multivariable Cox regression analysis for OS.

	UVA		MVA	
	HR (95% CI)	*p*	HR (95% CI)	*p*
Age (< 60/≥ 60, years)	1.083 (0.633–1.851)	0.772		
Gender (male/female)	0.850 (0.463–1.563)	0.602		
pTNM stage (I/II/III)^a^	2.569 (1.351–4.888)	**0.004**	2.457 (1.269–4.754)	**0.008**
Albumin (g/L)	0.973 (0.920–1.029)	0.340		
KPS score	0.945 (0.905–0.986)	0.090		
Pre‐WBC (×10^9^/L)	1.068 (0.979–1.165)	0.136		
Pre‐ALC (×10^9^/L)	1.500 (0.992–2.267)	0.055		
Pre‐ANC (×10^9^/L)	1.042 (0.943–1.151)	0.424		
Lymphopenia (I–III/IV)	1.815 (1.052–3.130)	**0.032**		
LRI	0.050 (0.007–0.360)	**0.003**	0.038 (0.004–0.323)	**0.003**
ΔALC	1.791 (1.203–2.666)	**0.004**		
Spleen V5 (cm^3^)	1.003 (1.000–1.005)	**0.032**		
Spleen V10 (cm^3^)	1.001 (1.000–1.002)	0.103		
Spleen V15 (cm^3^)	1.004 (1.001–1.007)	**0.021**		
Spleen V20 (cm^3^)	1.004 (1.000–1.008)	**0.037**		
Spleen V25 (cm^3^)	1.004 (0.999–1.008)	0.103		
Spleen V30 (cm^3^)	1.003 (0.998–1.009)	0.211		
Spleen V35 (cm^3^)	1.003 (0.996–1.010)	0.405		
Spleen V40 (cm^3^)	1.003 (0.994–1.012)	0.572		
Spleen V45 (cm^3^)	1.001 (0.987–1.015)	0.896		

Abbreviations: CI, confidence interval; HR, hazard ratio; MVA, multivariable analyses; OS, overall survival; UVA, univariable analyses; Vx, volume of spleen receiving × Gy of radiation. Bold values indicate statistical significance (*p* < 0.05).

^a^ According to the 8th edition of the American Joint Committee on Cancer system.

**TABLE 4:** Univariable and multivariable Cox regression analysis for DFS.

	UVA		MVA	
	HR (95% CI)	*p*	HR (95% CI)	*p*
Age (< 60/≥ 60, years)	1.396 (0.922–2.112)	0.115		
Gender (male/female)	0.818 (0.518–1.292)	0.389		
pTNM stage (I/II/III)^a^	1.682 (1.094–2.584)	**0.018**	1.640 (1.064–2.528)	**0.025**
Albumin (g/L)	0.947 (0.908–0.988)	0.110		
KPS score	0.978 (0.945–1.012)	0.201		
Pre‐WBC (×10^9^/L)	1.056 (0.985–1.132)	0.124		
Pre‐ALC (×10^9^/L)	1.378 (1.007–1.886)	**0.045**		
Pre‐ANC (×10^9^/L)	1.032 (0.954–1.117)	0.433		
Lymphopenia (I–III/IV)	1.424 (0.922–2.199)	0.111		
LRI	0.433 (0.165–1.138)	0.090		
ΔALC	1.515 (1.117–2.056)	**0.008**	1.482 (1.093–2.011)	**0.011**
Spleen V5 (cm^3^)	1.001 (0.999–1.004)	0.272		
Spleen V10 (cm^3^)	1.000 (0.999–1.001)	0.677		
Spleen V15 (cm^3^)	1.002 (0.999–1.005)	0.261		
Spleen V20 (cm^3^)	1.002 (0.998–1.005)	0.294		
Spleen V25 (cm^3^)	1.001 (0.997–1.005)	0.591		
Spleen V30 (cm^3^)	1.000 (0.996–1.004)	0.952		
Spleen V35 (cm^3^)	0.999 (0.993–1.004)	0.639		
Spleen V40 (cm^3^)	0.997 (0.990–1.004)	0.409		
Spleen V45 (cm^3^)	0.993 (0.981–1.005)	0.246		

Abbreviations: CI, confidence interval; DFS, disease‐free survival; HR, hazard ratio; MVA, multivariable analyses; UVA, univariable analyses; Vx, volume of spleen receiving × Gy of radiation. Bold values indicate statistical significance (*p* < 0.05).

^a^ According to the 8th edition of the American Joint Committee on Cancer system.

The 5‐year OS rate for patients with G4 lymphopenia (44.9 months vs. 62.9 months, *p* = 0.029) was worse compared to those without (Figure 3B). The spleen dosimetric parameters were also relatively higher in patients with G4 lymphopenia. Meanwhile, the ALC after CRT was relatively lower in these patients (Table 1).

### 3.4 Prognosis Affected by ΔALC and LRI During and After CRT

**FIGURE 4:**
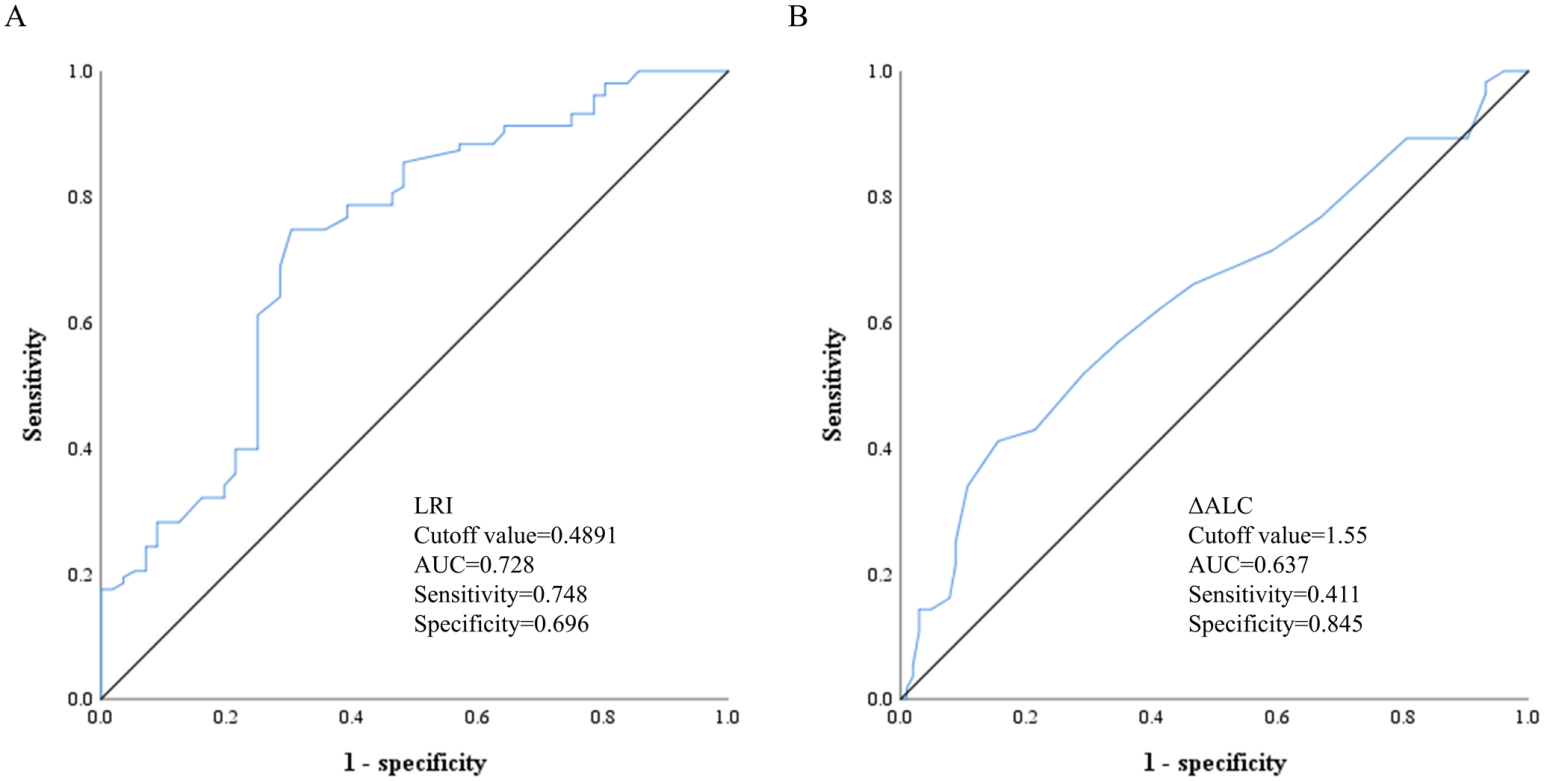
Predictive performance of LRI and ΔALC on prognosis. (A) Illustrated LRI related overall survival, cutoff value = 0.4891, AUC = 0.748. (B) Illustrated ΔALC related overall survival, cutoff value = 1.55, AUC = 0.637. ΔALC, changes in ALC; ALC, absolute lymphocyte count; LRI, lymphocyte recovery index.

At analysis, the median of LRI was 53.3% (range 7.0–200.0). As shown in Figure 4A,B, the enrolled patients were divided into four groups using ROC curves based on lymphocyte recovery status (LRI, cutoff point = 48.91%) and changes in ALC (ΔALC, cutoff point = 1.55): group A (ΔALC^hi^ → LRI^lo^) included patients with ΔALC ≥ 1.55 × 10^9^/L and LRI < 48.91% (*N* = 22, 13.8%), group B (ΔALC^hi^ → LRI^hi^) with ΔALC ≥ 1.55 × 10^9^/L and LRI ≥ 48.91% (*N* = 17, 10.7%). For patients with ΔALC < 1.55 × 10^9^/L, group C (ΔALC^lo^ → LRI^lo^) included those with LRI < 48.91% (*N* = 43, 27.1%), while group D (ΔALC^lo^ → LRI^hi^) with LRI ≥ 48.91% (*N* = 77, 48.4%).

**FIGURE 5:**
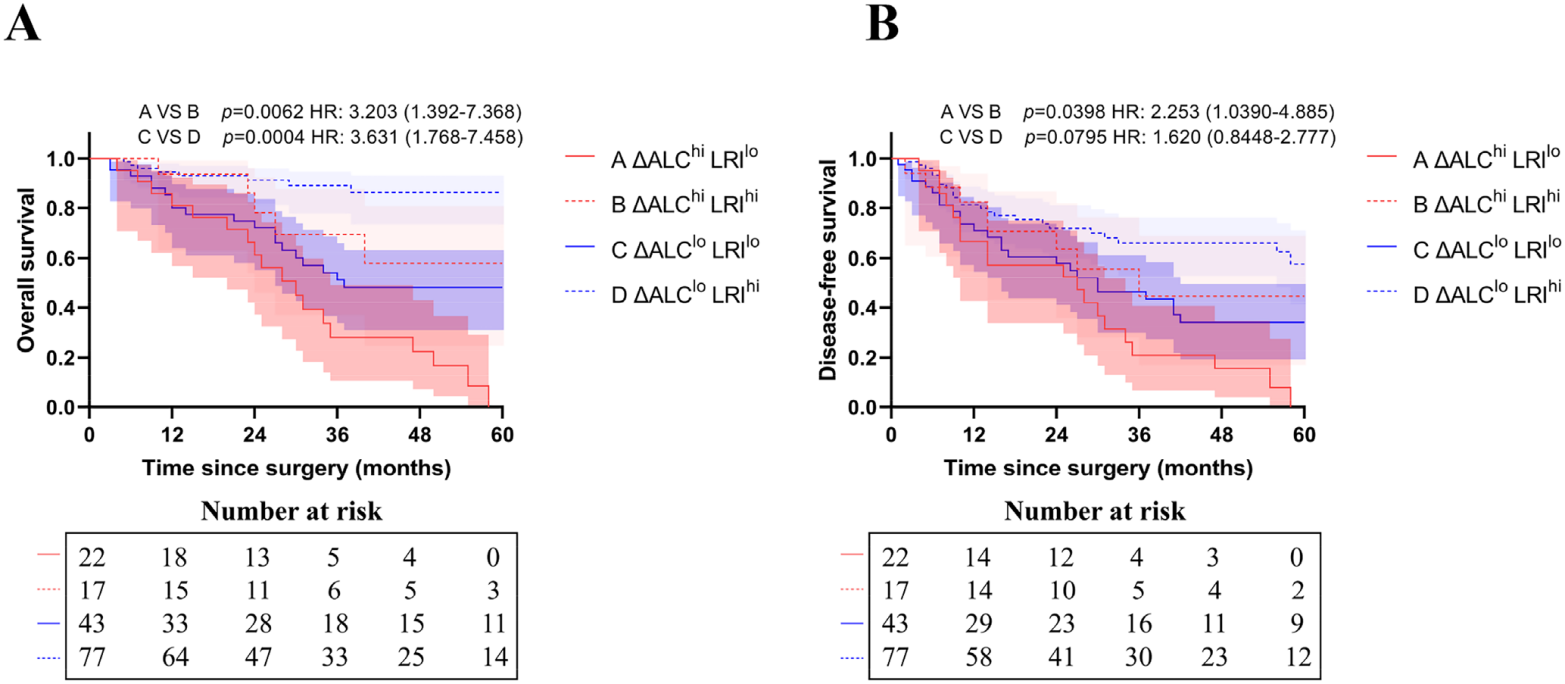
Kaplan Meier curves of (A) overall survival, (B) disease‐free survival between 4 groups. Group A (ΔALC^hi^ → LRI^lo^) included patients with ΔALC ≥ 1.55 × 10^9^/L and LRI < 48.91% (*N* = 22, 13.8%), group B (ΔALC^hi^ → LRI^hi^) with ΔALC ≥ 1.55 × 10^9^/L and LRI ≥ 48.91% (*N* = 17, 10.7%). For patients with ΔALC < 1.55 × 10^9^/L, group C (ΔALC^lo^ → LRI^lo^) included those with LRI < 48.91% (*N* = 43, 27.1%), while group D (ΔALC^lo^ → LRI^hi^) with LRI ≥ 48.91% (*N* = 77, 48.4%). *Time since surgery* refers to the time elapsed since R0/D1+ radical resection for gastric cancer in enrolled patients. The shaded area represents the 95% confidence interval of the curve. ALC, absolute lymphocyte count; HR, hazard ratio; LRI, lymphocyte recovery index.

As shown in Figure 5A,B, among patients with ΔALC ≥ 1.55 × 10^9^/L during CRT, group A had significantly worse 3‐year OS rate (28.1% vs. 72.8%, *p* = 0.0062) and 3‐year DFS rate (21.0% vs. 44.5%, *p* = 0.0398) compared to group B. For patients with ΔALC < 1.55 × 10^9^/L during CRT, survival curve analysis showed that group C had lower 3‐year OS (51.1% vs. 89.0%, *p* = 0.0004) and 3‐year DFS (46.3% vs. 65.9%, *p* = 0.0795) than group D.

## 4 Discussion

This retrospective study demonstrates the differences in lymphocyte reduction and recovery in GC patients receiving adjuvant CRT, which ultimately leads to variations in their prognosis. The dynamic changes in ALC can provide valuable indicators for clinical interventions. Immunotherapy has become an important method in cancer treatment, often used in combination with radiotherapy. Recent studies indicate that the depletion of immune cells after radiotherapy may reduce the benefits of immunotherapy in patients with non‐small cell lung cancer [14, 15].

Lymphocytes are highly sensitive to radiation due to their lack of effective DNA repair mechanisms. When lymphoid organs (such as bone marrow and spleen) or circulating lymphocytes are irradiated, it leads to a decrease in the circulating lymphocyte count [3, 16, 17]. This study showed that all patients' median of ALC (×10^9^/L) at baseline was 1.4, 25.2% of them had G1–3 lymphopenia prior to radiotherapy but no G4 lymphopenia; this may be related to chemotherapy. Several studies have shown that lower ALC at baseline is a negative predictor of patient prognosis across a range of cancer types [18, 19, 20, 21], but no such association was found in this study. During radiotherapy, the ALCs of all patients gradually decreased, reaching the lowest point at the sixth week of CRT, with a decrease of 85.71% compared with that before CRT, of which 69.8% and 30.2% of patients developed G1‐3 and G4 lymphopenia, respectively.

There is heterogeneity in lymphocyte decline in different tumors. Sun et al. [22] showed a regular decline in lymphocytes during both hypofractionated and conventional radiotherapy for breast cancer, with the incidence of lymphopenia was 45.4% and 55.7%, reaching a nadir at the end of radiotherapy and then beginning to recover 1 month after radiotherapy. The incidence of grade 3 lymphopenia is only 3%, and no patients experienced grade 4 lymphopenia.

Similar to previous studies, severe lymphopenia leads to a worse prognosis for patients. This study indicated that compared to patients with G1‐3 lymphocytopenia, G4 patients had a poorer 5‐year OS rate (44.9 vs. 62.9 months). While most previous studies across tumor types have similarly demonstrated that severe lymphopenia is associated with worse survival outcomes, some reports failed to identify a significant prognostic effect [23, 24, 25, 26]. Possible explanations for these discrepancies include differences in patient characteristics (e.g., tumor type and stage), variations in treatment modalities (such as radiotherapy techniques and concurrent chemotherapy regimens), and heterogeneity in the timing and definition of lymphopenia/recovery assessment. These factors may account for the inconsistent findings across studies. Importantly, our results provide additional evidence supporting lymphopenia as a clinically relevant prognostic factor. In addition to the pTNM stage, LRI and ΔALC were another factor affecting OS and DFS respectively. The LRI is a comparison of the lymphocyte count at a certain time after CRT with the baseline before CRT, representing the ability to recover after lymphocyte decline. A study on esophageal cancer showed that insufficient lymphocyte recovery (LRI < 60%) was significantly associated with poor prognosis [12]. In our study, patients with LRI < 48.91% were regarded as insufficient lymphocyte recovery. Although previous studies have used the nadir ALC to quantify radiation‐related immune system damage [27], it is significantly influenced by the patient's baseline. As a result, some alternative indicators have been proposed, such as lymphocyte exhaustion kinetics [27, 28], logarithmic lymphocyte depletion [29], or lymphocyte recovery at 3–6 months posttreatment [11, 12].

The relationship between lymphocyte recovery and prognosis after radiotherapy remains unclear. Studies on pancreatic cancer and lung cancer have shown a significant correlation between lymphocyte recovery 3–6 months post‐CRT and better prognosis [11, 30]. Additionally, Lee et al. [30] found that lower baseline ALC and larger planning target volume are associated factors for lymphopenia. However, in esophageal cancer, lymphocyte recovery 6–8 weeks post‐CRT did not correlate with long‐term prognosis [13]. Recovery of lymphocyte count can be a long‐term process, which may depend on the damage to lymphocytes and other organs at different sites of tumor treatment.

We grouped the patients based on the degree of lymphocyte depletion and recovery to compare their survival outcomes. Our results revealed that the more decline during CRT and inadequate recovery of lymphocytes at 120 days after CRT were significantly associated with poorer OS and DFS. Therefore, we recommend focusing on lymphocyte recovery after CRT and implementing interventions within a few weeks post‐CRT to promote recovery, while also preventing lymphopenia during CRT.

The immune systems related to RIL can be divided into two categories: one is the system that circulates through the irradiated field, such as the lungs, heart, and large blood vessels; the other is responsible for lymphocyte development and hematopoietic functions, such as the spleen, bone marrow, and thymus. In the treatment planning of the former, selecting an appropriate irradiated area, reducing low‐dose scatter, and limiting the dose to the latter may help reduce the incidence of lymphopenia. As previous studies have shown, the lung V_5_ and D_mean_ are dose parameters associated with the incidence of lymphopenia [31, 32]. A systematic review including 14 studies pointed out that tumor volume, lung V_5_, and heart V_5_ were predictive factors for lymphopenia after RT in lung cancer [33]. Huang et al. found that G3‐4 lymphopenia was associated with brain V_25_ > 60%, and this was correlated with a lower overall survival in high‐grade gliomas [34].

Our results showed that the splenic D_mean_ and V_5_ were related to G4 lymphopenia, and splenic V_5_–V_45_ was always related to LRI. As the splenic hilum was often included in the clinical target volume of the gastric cancer, the spleen would be involved in the irradiation field to varying degrees inevitably, although it contains a large number of lymphocytes. In postoperative adjuvant chemoradiotherapy, the splenic dose is usually very high; our result showed the median splenic mean dose is 25.94 Gy, and the maximum is 41.39 Gy. Our study indicated that serum albumin levels and KPS scores do not show a significant correlation with the LRI. This observation suggests that these factors, often regarded as indicators of nutritional status and overall physical health, may not independently impact lymphocyte recovery and patient prognosis. Other factors, such as baseline immune function and splenic dosimetric parameters, may play a more crucial role in influencing LRI. These findings highlight the complexity of the immune recovery process, and considering the uncertainties associated with pre‐ALC, controlling splenic dosimetric parameters remains one of the few feasible options for impacting lymphocyte recovery.

The spleen has a rich blood supply, and the lymphocytes in the blood must pass through tortuous microvessels to re‐enter the circulation, which leads to a significant increase in both the number of irradiated lymphocytes and the duration of exposure [6]. However, the spleen has not been considered an OAR and is not routinely delineated in radiotherapy, with limited data available on the hematological toxicity of spleen irradiation. In abdominal tumor radiotherapy, unintentional exposure of the spleen may have a different impact on lymphocytes compared to the irradiation of other lymphoid organs, large blood vessels, or the heart.

Trip et al. revealed the dose‐effect relationship of the spleen after CRT for gastric cancer, suggesting that the spleen's tolerable dose to maintain normal immune function should be at least below 12 Gy [35]. Chadha et al. found that higher spleen doses increase the risk of severe post‐CRT lymphopenia in pancreatic cancer patients, with a D_mean_ of 9 Gy recommended as the dose limit for RT plans [7]. A retrospective study on esophageal cancer revealed that spleen dose‐volume parameters (such as V_5_, V_10_, V_20_, V_30_, and D_mean_) are independent factors leading to a decrease in the nadir ALC. For every 1 Gy increase in D_mean_, the nadir ALC decreases by 2.9% [36]. Another regression analysis on abdominal tumors indicated that for every 1 Gy increase in D_mean_, the probability of developing G3‐4 lymphopenia increases by 18.6% [37]. Similar findings have shown that a 1 Gy increase in D_mean_ is associated with a 1% decrease in nadir ALC, and unintended spleen irradiation (including V_15_ and D_max_) is associated with lymphopenia during CRT for abdominal tumors [38].

In radiotherapy planning for abdominal tumors, minimizing splenic irradiation is essential to preserve lymphocytes. Our study identified that maintaining spleen V_5_ below 180.6 cm^3^ could prevent grade 4 lymphopenia, while limiting V_5_ to less than 272.2 cm^3^ was associated with a 60.9% reduction in death risk. These findings are consistent with the retrospective study by Liu et al. in hepatocellular carcinoma [39], supporting the importance of restricting low‐dose splenic exposure. Among various dose–volume parameters (V_5_–V_45_), the D_mean_ and low‐dose volume (V_5_) emerged as independent predictors of severe lymphopenia. Clinically, both are straightforward to calculate during routine treatment planning: V_5_ reflects the extent of low‐dose exposure to lymphocyte‐rich compartments, whereas D_mean_ represents the cumulative radiation burden. Therefore, prioritizing V_5_ and D_mean_ as practical dose constraints may represent the most relevant strategy to mitigate severe lymphopenia and improve patient outcomes in abdominal radiotherapy.

This study has the following limitations. First, the sample size of patients is limited, and the chemotherapy regimens varied. Additionally, due to limited follow‐up data, there is no data on lymphocyte recovery beyond 6 months. Finally, although different lymphocyte subgroups play distinct roles in immune responses, this study did not identify them. However, this study was one of the few to investigate how the dynamics of lymphocyte depletion and recovery during and after gastric cancer CRT affected the prognosis, and how splenic dosimetry parameters affected lymphopenia and provided recommended dosimetric limits.

## 5 Conclusions

Lymphocyte count decline and insufficient recovery during and after gastric cancer CRT induced the worse prognosis in patients. Promoting lymphocyte recovery after CRT is equally important as preventing lymphocyte depletion during CRT. Splenic D_mean_ and V_5_ were related to G4 lymphopenia and eventually affect prognosis, constraining the spleen V_5_ to < 180.6 cm^3^ and < 272 cm^3^ may respectively improve G4 lymphopenia and further reduced the risk of death by 60.9%.

## Author Contributions

**Yifu Ma:** investigation, data curation, and writing – original draft; **Shuying Zhang:** investigation, and writing – review and editing. **Jiayan Ma:** data curation. **An Gao:** formal analysis. **Jiale Liu:** data curation. **He Ma:** visualization. **Qiyi Zhou:** data curation. **Jianjun Qian:** resources, data curation, and supervision; **Liyuan Zhang:** conceptualization, methodology, and supervision.

## Funding

This work was supported by the National Natural Science Foundation of China (Grant No. 82573449); the Pioneer Talent Program of the Second Affiliated Hospital of Soochow University (Grant No. XKTJ‐RC202401); the Suzhou Major Disease Multicenter Clinical Research Project (Grant No. DZXYJ202304); and the Internal Collaborative Research Program of the State Key Laboratory of Radiation Medicine and Radiation Protection (jointly established by the Ministry of Science and Technology and Jiangsu Province) (Grant No. GZN1202302).

## Ethics Statement

Our retrospective study abided by the rules of medical ethics. The study was approved by the ethics committee of The Second Affiliated Hospital of Soochow University. Written informed consent was obtained from all patients for using their data.

## Conflicts of Interest

The authors declare no conflicts of interest.

## Supporting information


**Appendix S1:** cam471553‐sup‐0001‐AppendixS1.docx.

## Data Availability

The data that support the findings of this study are available from the corresponding author upon reasonable request.
