# Supplementary material for: Clinical Considerations of Splenic Dose Constraints to Mitigate Radiation‐Induced Lymphopenia
Source: Cancer Med. 2026 Jan 22;15(1):e71553. doi: 10.1002/cam4.71553 (PMC12824635; doi:10.1002/cam4.71553)
Supplement: Supplementary file 1 — Appendix S1: cam471553‐sup‐0001‐AppendixS1.docx. [file CAM4-15-e71553-s001.docx]

**Table S1**

Selective lymph node station irradiation of different primary tumor sites.

| Primary tumor location | Selected regional lymph nodes |
| --- | --- |
| Upper 1/3 stomach and distal EGJ | No. 110, No. 20, No. 1-3, No. 7-12, No. 16a |
| Middle 1/3 stomach | No. 1-3, No. 5-13, No. 14*, No. 16a |
| Lower 1/3 stomach | No. 3, No. 5-9, No. 11p, No. 12-13, No. 14*, No. 16a |

EGJ: esophagogastric junction.

*When No. 12-13 lymph node metastasis or pancreatic invasion.

**Table S2**

The *P* value of cross-correlation analysis among the dosimetric parameters.

|  | **Dmax** | **Dmean** | **V5** | **V10** | **V15** | **V20** | **V25** | **V30** | **V35** | **V40** | **V45** |
| --- | --- | --- | --- | --- | --- | --- | --- | --- | --- | --- | --- |
| **Dmax** | 0 | 0 | 0.005 | 0.003 | 0 | 0 | 0 | 0 | 0 | 0 | 0 |
| **Dmean** | 0 | 0 | 0.644 | 0.051 | 0 | 0 | 0 | 0 | 0 | 0 | 0 |
| **V5** | 0.005 | 0.644 | 0 | 0 | 0 | 0 | 0 | 0 | 0 | 0 | 0 |
| **V10** | 0.003 | 0.051 | 0 | 0 | 0 | 0 | 0 | 0 | 0 | 0 | 0 |
| **V15** | 0 | 0 | 0 | 0 | 0 | 0 | 0 | 0 | 0 | 0 | 0 |
| **V20** | 0 | 0 | 0 | 0 | 0 | 0 | 0 | 0 | 0 | 0 | 0 |
| **V25** | 0 | 0 | 0 | 0 | 0 | 0 | 0 | 0 | 0 | 0 | 0 |
| **V30** | 0 | 0 | 0 | 0 | 0 | 0 | 0 | 0 | 0 | 0 | 0 |
| **V35** | 0 | 0 | 0 | 0 | 0 | 0 | 0 | 0 | 0 | 0 | 0 |
| **V40** | 0 | 0 | 0 | 0 | 0 | 0 | 0 | 0 | 0 | 0 | 0 |
| **V45** | 0 | 0 | 0 | 0 | 0 | 0 | 0 | 0 | 0 | 0 | 0 |

**Table S3**

Interaction analysis of grade 4 lymphopenia during chemoradiotherapy

|  | OR (95% CI) | *P* value |
| --- | --- | --- |
| Maximum spleen dose*chemotherapy regimen | 1.002 (0.997-1.007) | 0.400 |
| Mean spleen dose*chemotherapy regimen | 1.001 (0.999-1.003) | 0.206 |
| Spleen V5*chemotherapy regimen | 1.025 (0.990-1.060) | 0.160 |
| Spleen V10*chemotherapy regimen | 0.932 (0.609-1.424) | 0.744 |
| Spleen V15*chemotherapy regimen | 1.083 (0.986-1.189) | 0.095 |
| Spleen V20*chemotherapy regimen | 1.005 (0.944-1.070) | 0.876 |
| Spleen V25*chemotherapy regimen | 0.988 (0.922-1.058) | 0.721 |
| Spleen V30*chemotherapy regimen | 1.012 (0.923-1.108) | 0.806 |
| Spleen V35*chemotherapy regimen | 0.952 (0.830-1.092) | 0.482 |
| Spleen V40*chemotherapy regimen | 1.052 (0.900-1.229) | 0.527 |
| Spleen V45*chemotherapy regimen | 0.979 (0.885-1.083) | 0.682 |

OR: odds ratio; CI: confidence interval; Vx: volume of spleen receiving x Gy of radiation.

**Table S4**

Interaction analysis of grade 4 lymphopenia post chemoradiotherapy

|  | OR (95% CI) | *P* value |
| --- | --- | --- |
| Maximum spleen dose*chemotherapy regimen | 1.000 (0.999-1.001) | 0.420 |
| Mean spleen dose*chemotherapy regimen | 1.000 (0.998-1.002) | 0.981 |
| Spleen V5*chemotherapy regimen | 1.000 (0.982-1.018) | 0.969 |
| Spleen V10*chemotherapy regimen | 0.942 (0.861-1.031) | 0.194 |
| Spleen V15*chemotherapy regimen | 1.097 (0.986-1.219) | 0.089 |
| Spleen V20*chemotherapy regimen | 0.997 (0.940-1.058) | 0.925 |
| Spleen V25*chemotherapy regimen | 0.957 (0.891-1.028) | 0.233 |
| Spleen V30*chemotherapy regimen | 1.077 (0.969-1.197) | 0.167 |
| Spleen V35*chemotherapy regimen | 0.958 (0.814-1.126) | 0.601 |
| Spleen V40*chemotherapy regimen | 0.970 (0.790-1.192) | 0.775 |
| Spleen V45*chemotherapy regimen | 1.001 (0.865-1.158) | 0.991 |

OR: odds ratio; CI: confidence interval; Vx: volume of spleen receiving x Gy of radiation.

**Table S5**

Logistic analysis for the patients between LRI^hi^ group and LRI^lo^ group.

|  | UVA | |
| --- | --- | --- |
|  | OR (95% CI) | *P* value |
| Age (<60/≥60, years) | 1.254 (0.656-2.398) | 0.494 |
| Gender (Male/Female) | 0.992 (0.476-2.068) | 0.983 |
| pTNM stage (I/II/III) * | 1.596 (0.821-3.100) | 0.168 |
| Albumin (g/L) | 0.994 (0.932-1.059) | 0.843 |
| KPS score | 0.987 (0.940-1.035) | 0.582 |
| Pre-ALC (×10^9^/L) | 2.125 (1.180-3.826) | 0.012 |
| Spleen V5 (cm^3^) | 1.004 (1.000-1.007) | 0.047 |
| Spleen V10 (cm^3^) | 1.005 (1.001-1.009) | 0.016 |
| Spleen V15 (cm^3^) | 1.006 (1.001-1.010) | 0.014 |
| Spleen V20 (cm^3^) | 1.006 (1.001-1.012) | 0.013 |
| Spleen V25 (cm^3^) | 1.007 (1.001-1.013) | 0.014 |
| Spleen V30 (cm^3^) | 1.009 (1.002-1.016) | 0.013 |
| Spleen V35 (cm^3^) | 1.011 (1.002-1.020) | 0.018 |
| Spleen V40 (cm^3^) | 1.016 (1.003-1.029) | 0.014 |
| Spleen V45 (cm^3^) | 1.036 (1.011-1.062) | 0.004 |

LRI: lymphocyte recovery index; UVA: univariable analyses; OR: odds ratio; CI: confidence interval; Vx: volume of spleen receiving x Gy of radiation.

* According to the 8th edition of the American Joint Committee on Cancer system.





**Figure S1** Flowchart of patient inclusion and exclusion based on eligibility criteria.





**Figure S2** The dynamic changes of white blood cells and neutrophils from baseline through week 1-6 and 120 days after the end of CRT, represented by median value with quartiles.


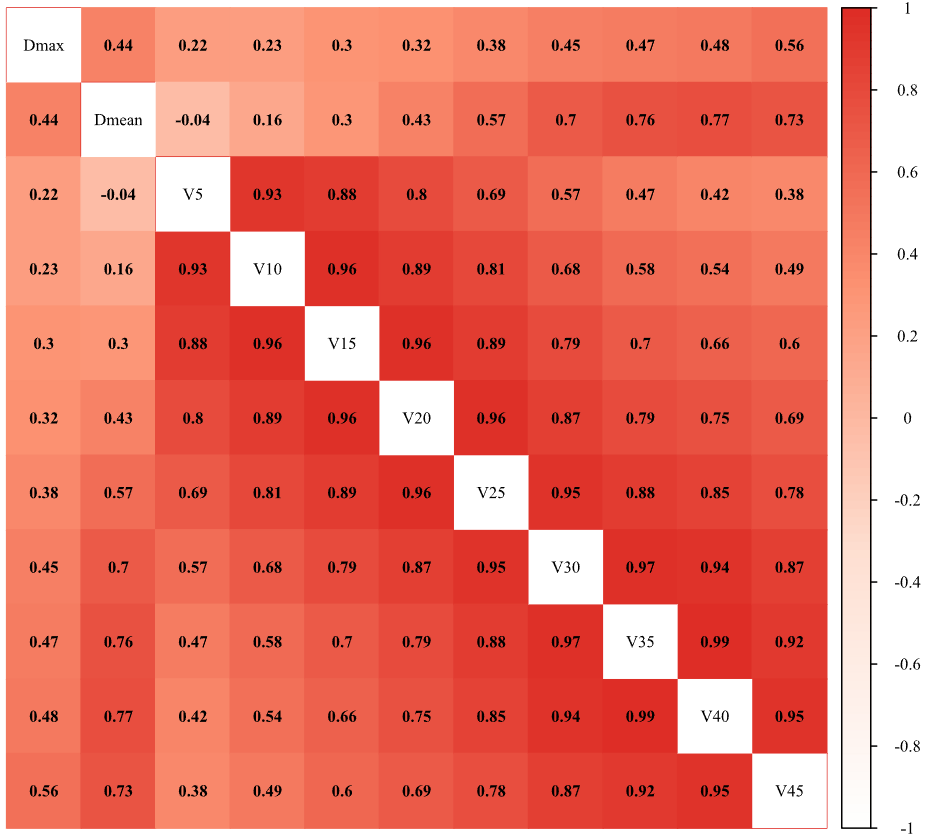


**Figure S3** The correlation coefficient of cross-correlation analysis among the dosimetric parameters.
